# Transcriptionally regulated miR-26a-5p may act as BRCAness in Triple-Negative Breast Cancer

**DOI:** 10.1186/s13058-023-01663-y

**Published:** 2023-06-26

**Authors:** Yue Zhang, Lianqiu Lv, Renjing Zheng, Rong Xie, Yuanhang Yu, Han Liao, Jianying Chen, Bo Zhang

**Affiliations:** 1 Department of Breast and Thyroid Surgery, Union Hospital, Tongji Medical College, Huazhong University of Science and Technology, Wuhan, 430022 China; 2 Department of Gastrointestinal Surgery, Union Hospital, Tongji Medical College, Huazhong University of Science and Technology, Wuhan, 430022 China

**Keywords:** TNBC, miR-26a-5p, DNA damage, HRD, Cisplatin, Synthetic lethal

## Abstract

**Background:**

DNA damage and DNA damage repair (DDR) are important therapeutic targets for triple-negative breast cancer (TNBC), a subtype with limited chemotherapy efficiency and poor outcome. However, the role of microRNAs in the therapy is emerging. In this study, we explored whether miR-26a-5p could act as BRCAness and enhance chemotherapy sensitivity in TNBC.

**Methods:**

Quantitative reverse transcription polymerase chain reaction (RT-qPCR) was used to detect the expression of miR-26a-5p in breast cancer tissues and cell lines. CCK-8 was used to measure drug sensitivity in concentration gradient and time gradient. Comet assay was used to detect DNA damage. Flow cytometry was performed to examine apoptosis. Moreover, we used western blot and immunofluorescence to detect biomarkers. Luciferase reporter assay was performed to verify the combination of miR-26a-5p and 3’UTR of target gene. Hormone deprivation and stimulation assay were used to validate the effect of hormone receptors on the expression of miR-26a-5p. Chromatin immunoprecipitation (ChIP) assays were used to verify the binding sites of ER-a or PR with the promoter of miR-26a-5p. Animal experiments were performed to the effect of miR-26a-5p on Cisplatin treatment.

**Results:**

The expression of miR-26a-5p was significantly downregulated in TNBC. Overexpressing miR-26a-5p enhanced the Cisplatin-induced DNA damage and following apoptosis. Interestingly, miR-26a-5p promoted the expression of Fas without Cisplatin stimulating. It suggested that miR-26a-5p provided a hypersensitivity state of death receptor apoptosis and promoted the Cisplatin sensitivity of TNBC cells in vitro and in vivo. Besides, miR-26a-5p negatively regulated the expression of BARD1 and NABP1 and resulted in homologous recombination repair defect (HRD). Notably, overexpressing miR-26a-5p not only facilitated the Olaparib sensitivity of TNBC cells but also the combination of Cisplatin and Olaparib. Furthermore, hormone receptors functioned as transcription factors in the expression of miR-26a-5p, which explained the reasons that miR-26a-5p expressed lowest in TNBC.

**Conclusions:**

Taken together, we reveal the important role of miR-26a-5p in Cisplatin sensitivity and highlight its new mechanism in DNA damage and synthetic lethal.

**Supplementary Information:**

The online version contains supplementary material available at 10.1186/s13058-023-01663-y.

## Background

Breast cancer is the most commonly diagnosed cancer worldwide, and it is the first leading cause of cancer-related death in women [1]. Based on the expression status of the estrogen receptor (ER-a), progesterone receptor (PR) and human epidermal growth factor 2 receptor (HER2/ERBB2), breast cancer is classified as luminal A, luminal B, HER2 positive and TNBC [2]. TNBC is characterized by the absence of the three targetable receptors, and thus, patients of TNBC show low chemotherapy response and poor outcome [3].

MicroRNAs are small non-coding RNA which mostly combine the 3’ untranslated region (UTR) or rarely combine the 5’UTR of a target mRNA to regulate post-transcriptional modification and mediate cancers’ apoptosis, proliferation and so on [4]. MiR-26a-5p, also called miR-26a-1, has been previously demonstrated that it plays an anti-oncogene role in tumor’s tumor proliferation, metastasis and apoptosis, such as renal cell carcinoma, hepatocellular carcinoma, gastric cancer and breast cancer [5–8]. However, the underlying mechanism of the miR-26a-5p in DNA damage repair has not yet been explored.

Cisplatin is the first generation of platinum drug which interacts with both intra- and inter-strand DNA cross-links (ICLs) to stall replication forks to kill cancer cells [9]. ICLs triggers a complex intracellular signal transduction cascade to active DDR to repair the lesions including single-strand DNA (ssDNA) errors and double-strand breaks (DSBs), such as nucleotide excision repair (NER), mismatch repair (MMR), homologous recombination (HR) and non-homologous end joining (NHEJ) [10–12]. DSBs are the most dangerous among all types of DNA damage. Notably, the HR repair (HRR) pathway is a highly conserved manner that ensure the accurate repair of DSBs by using the intact sister chromatid as a template for repair, thereby maintaining the sequence integrity [13]. Therefore, the dysfunction of HR-related genes lead to genomic instability, which is a hallmark of cancer [14].

BARD1, the chaperone of BRCA1 translocation into and retention in the nucleus, is an E3 ubiquitin-protein ligase essential for BRCA1 stability. ATM kinase phosphorylated BRCA1 is recruited to DNA damage sites and then binds with the BARD1 to form BARD1-BRCA1complex [13, 15]. The complex, the key component of HR, plays a vital role in the DSB repair [16]. NABP1, a member of single-stranded DNA binding proteins (SSBs) also called hSSB2 or OBFC2A, is the close homolog of hSSB1 which stimulates the activity of RAD51 recombinase and/or by recruiting RAD51, a biomarker of HRR, to the lesions. They form different hSSB complexes which are required for HR-dependent DNA repair and maintenance of genome stability [17–19]. H2AX is phosphorylated by ATM kinase at sites of DNA lesions as γH2AX, a marker of DNA damage, enabling DNA repair proteins are recruited to DNA damage sites [20]. Ultimately, if DDR fails to remove the lesions, the specific DNA lesions formed blockage of DNA replication which leads to the collapse of replication forks will trigger apoptosis [21, 22].

The lack of effective chemotherapies has forested a major effort to discover more targetable molecular targets to treat TNBC exactly as synthetic lethality [23]. For example, germline BRCA1/2 mutations (gBRCAm) or ‘BRCAness’ result in HR deficiency making the tumors sensitive to poly-(ADP ribose)-polymerase inhibitors (PARPis) because they have a specific type of DNA repair defect. PARPis cause persistent SSBs which potentially create DSBs if they encountered by replication forks then resulting in the collapse of the forks. PARP1is trap PARP1 on DNA, preventing autoPARylation and PARP1 release, therefore arresting the catalytic cycle of PARP1. The greater efficiency of inhibiting PARP-mediated repair of DNA damage induced by chemotherapy or radiotherapy could be achieved [24]. It has been revealed that PARP1is play important roles in DDR, transcriptional regulation and cell apoptosis and exhibited huge potency in cancer therapy. So far, there were four PARPis approved for the treatment of several cancers, including Olaparib. Recent studies have significantly broadened the concept of BRCAness that it is not only a defect in mimicking BRCA1 or BRCA2 loss but also defects in HRR, replication fork protection and the subsequent hypersensitivity to DNA damaging agents. BRCAness is used to predict responses to agents as PARP1is or platinum-based salts in patients without gBRCAm [25–27].

ER-a and PR belong to the nuclear receptor family and play diverse roles in biological processes via transcriptional regulating [28]. In breast cancer, the two receptors are biomarkers for classification and targets for endocrine therapy. However, whether the loss of the two genes affects the expression of downstream genes that participate in the progress of TNBC remains unknown.

In this study, we demonstrated that miR-26a-5p acts as BRCAness via negatively regulating BARD1/NABP1 and providing a potential therapeutic strategy. Besides, we explored the mechanism that miR-26a-5p is especially downregulated in TNBC.

## Methods

### miRNA-seq analysis

The miRNA-seq data of breast cancer were downloaded from The Cancer Genome Atlas (TCGA). The packages of GDCRNATools, LIMMA, edgeR and ggplot2 for R were used to analyze miRNA-seq data.

### Clinical samples

Total 51 paired samples of human breast cancer tissue and their matched adjacent normal tissue were collected from Wuhan Union Hospital, Tongji medical college, Huazhong University of Science and Technology (Wuhan, China), between 2016 and 2019.

### Cell culture

MCF-7 and 293T cell lines were cultured in DMEM supplemented with 10% fetal bovine serum (FBS). T47D, BT549, MDA-MB-468, BT474 cell lines were cultured in Roswell Park Memorial Institute-1640 (RPMI-1640, Gibco) supplemented with 10% FBS. BT20 cells were cultured in MEM (Gibco) supplemented with extra 1% non-essential amino acid (NEAA) and 10% FBS. MCF10A cells were cultured in DMED/F12 supplemented with extra 10 µg/ml insulin, 20 ng/ml epidermal growth factor (EGF), 100 µg/ml cholera toxin, 0.5 µg/ml hydrocortisone and 5% FBS. The above cell lines were all cultured in 5%CO_2_ environment. MDA-MB-231 cell lines were cultured in L15 supplemented with 10% FBS in a CO2 free environment.

### RNA extraction and RT-qPCR

Total RNA was obtained with RNAiso for Small RNA regent (Takara) from cell lines and frozen fresh tissue. cDNAs were reverse transcribed with PrimeScript™ RT reagent Kit (Takara). RT-qPCR was performed with the TB Green® Premix Ex Taq™ (Takara) on the Bio-Rad CFX96 Touch Deep Well Real-Time PCR Detection System. Specific primer pairs of miRNA-26a-5p were purchased from RiboBio (Guangzhou, China). And relative expression values for miRNA-26a-5p were obtained by normalizing to the expression of U6 gene using the ΔΔCt method.

### Transfection

The miR-26a-5p mimic and siRNA for NABP1 were purchased from RiboBio (Guangzhou, China). The shRNAs for BARD1 and overexpressing plasmids for BARDA, NABP1, ER-a, PR were purchased from GENECHEN (Shanghai, China). Cells were transfected with siRNA, plasmids and mimic using Lipofectamine 3000 reagent (Invitrogen) according to the protocol.

### CCK8 assay

Cells were seeded in 96-well plates at 2500–3000 cells per well and treated with different concentrations of Cisplatin (Selleck) with medium containing 1% FBS. After 72 h of treatment, Cell Counting Kit-8 (CCK8, Bimake) was added at one-tenth of the volume of the wells’ medium. After 2 h, the OD value of per well was measured at 450 nm with a Bio-Rad iMark spectrometer.

### Comet assay

Alkaline comet assays were performed to detect both single-strand break (SSB) and double-strand break (DSB). According to the quick protocol, 500 µl of low melt agarose (LMAgarose) was added to 5000 cells in 50 µl of PBS and pipetted onto Trevigen® comet slides. Once gel had solidified, slides were incubated in lysis solution overnight at 4 °C. After incubated in 4 °C for 1 h with alkaline unwinding solution (0.6 g NaOH, 250 µl 200 mM EDTA, 49.75 ml ddH2O), electrophoresis was performed for 30 min at 23 V in alkaline electrophoresis solution (300 mM NaOH, 1 mM EDTA). After washes, slides were stained with 100 µl 0.01% SYBR®Gold. Fifty cells from each condition were evaluated with the CASP Comet analysis system from pictures obtained with an electron fluorescence microscope.

### Immunofluorescence

The treated cell slides were washed with PBS in the culture plate, then fixed with 4% paraformaldehyde for 15 min and permeabilized with 0.5% Triton X-100 at room temperature for 20 min after washing with PBS. After washing 3 times with PBS, normal goat serum was added to the glass slides and blocked at room temperature for 30 min. The blocking solution was removed by absorbent paper, and then, the primary antibody was added dropwise. And the cells were incubated at 4 °C overnight. Incubate the fluorescent secondary antibody under the dark condition at room temperature for 1 h or 37 °C for 30 min. After washing with PBS, add DAPI and incubate in the dark for 5 min to stain the specimen. Block slides with mounting fluid containing anti-fluorescence quencher. Finally, the collected images were observed under a fluorescence microscope.

### Luciferase reporter assay

The BARD1 3’UTR and NABP1 3’UTR (WT) or mutant (MUT) plasmids were purchased from RiboBio (Guangzhou, China). The luciferase reporter assay was performed with the Dual-Glo® Luciferase Assay System (Promega) according to the protocol.

### Flow cytometric analysis

Cells were plated 24 h prior to treatment with Cisplatin. After treatment, medium was collected separately to stop the reaction of free-EDTA trypsin and cells. All cells were collected. After washes with PBS, 500 µl binding buffer resuspended the cells per cube. After incubated with 5 µL Annexin-V FITC and 5 µl 7-ADD in the dark environment at room temperature for 5–15 min, flow cytometry detection was performed.

### Western blot analysis

SDS–polyacrylamide gel electrophoresis was performed using 12% bis–tris gels (Biosharp), and proteins were transferred to PVDF membranes by semidry transfer using Trans-Blot transfer medium (Bio-Rad). Membranes were blocked in 5% skimmed milk in TBS-T and incubated overnight at 4 °C with primary antibodies, including γH2AX (#7918, Cell Signaling Technology), Caspase-3 (#14220, Cell Signaling Technology), Caspase-9(#9508, Cell Signaling Technology), Bax (#5023, Cell Signaling Technology), Bcl-2(#15071, Cell Signaling Technology), ER-a (#8644, Cell Signaling Technology), PR (#8757, Cell Signaling Technology), RAD51(14961-1-AP, Proteintech), Fas (ab133619, Abcam), FADD (ab124812, Abcam).

### Hormone deprivation and stimulation assay

T47D and MCF-7 cell lines were cultured in phenol red-free RPMI-1640 (BOSTER, China) or phenol red-free DMEM (BOSTER, China) supplemented with 10% dextran charcoal-stripped bovine serum (Biological Industries, China) for 48 h. After hormone deprivation, β-Estradiol (E2, Sigma, E2758) or Etonogestrel (Selleck, S4673) was added to the free-hormone medium in different concentrations for next cell culture.

### ChIP assay

Chromatin immunoprecipitation assays were performed with SimpleChIP® Plus Enzymatic Chromatin IP Kit (Cell Signaling Technology). Immunoprecipitation was performed with anti-ER-a (#8644, Cell Signaling Technology) and anti-PR antibodies (#8757, Cell Signaling Technology). Specific regions were quantified via qRT-PCR using the primers:

PR Bindsite: Sense primer 5′–AGGCTGAGGAGGCACTTTGT–3′, Anti-sense primer 5′–AGTGGGCATTTTCGGGTG–3′.

ER-a Bindsite1: Sense primer 5′–CCCTTCCGAATCCTTCCAGTG–3′, Anti-sense primer 5′–TCGCCTGGTGGGGGAGA–3′;

Bindsite2: Sense primer 5′–CTCTGCCGTCCGCTACACC–3′, Anti-sense primer 5′–GGAAGGAGAAAGGAAGGGAGG–3′;

Bindsite3: Sense primer 5′–CGCCCTCGCTCGCTCCTT–3′, Anti-sense primer 5′–GCCCCCCGCAAGCCAA–3′.

### Viral infection and animal experiments

To obtain stable miRNA-26a-5p-expressing MDA-MB-231 cell line for in vivo study, cells were infected with hU6-MCS-EGFP-miRNA-26a-5p lentivirus (GENECHEM, China). Four-week-old female BALB/c nude mice were purchased from Vital River for the in vivo study. The mice were injected subcutaneously around their second mammary gland with 1 × 10^7^ cells in 100 µl free-FBS L15 mixed with 100 µl Matrigel matrix (Corning, #354234). Tumor volume (TV) (mm^3^) = L × W^2^ × π/6 , where L is the length and W is the width. Relative tumor volume (RTV) = Vt/V_0_, where Vt is the recorded volume after treatment and V_0_ at the start of treatment. The relative tumor growth inhibition T/C ratio is used to evaluate the efficacy of drugs in tumor xenograft experiments by the following formula: T/C% = Treatment-RTV/Control-RTV × 100%. Cisplatin (2.5 mg/kg) or ddH2O was peritoneal injected when one group’s volume exceeded 150 mm^3^.

### Statistical analysis

All data are presented as mean values ± SD. And all the statistical analyses were performed using SPSS software. The differences were assessed using two-tailed Student’s *t* test for group comparisons. All in vitro experiments were conducted three times. Differences were considered significant when *P* value was less than 0.05. *P < 0.05, **P < 0.01, ***P < 0.001, ****P < 0.0001 versus control. N.S. not significant.

## Results

### miR-26a-5p is downregulated in TNBC

**Fig. 1:**
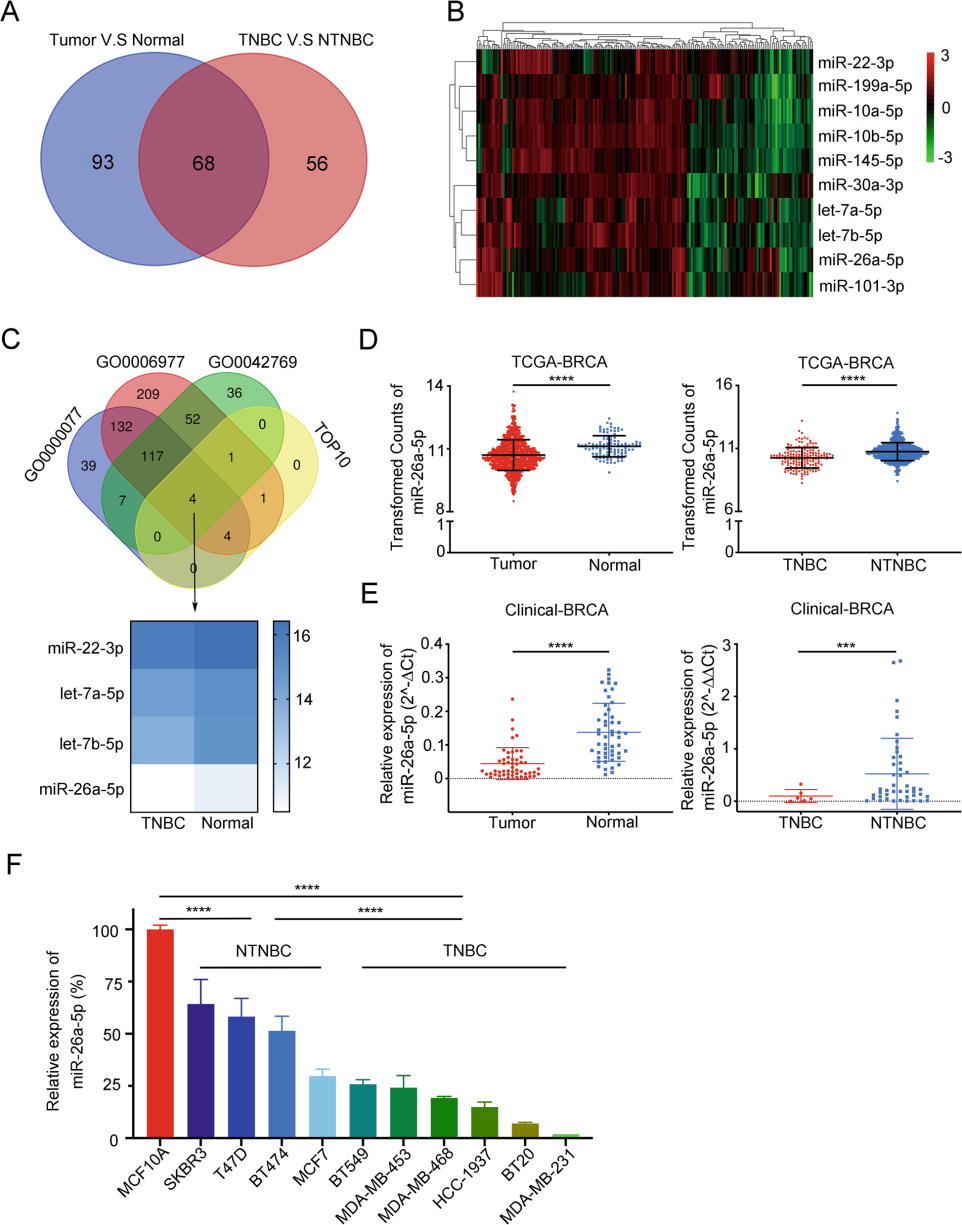
miR-26a-5p is downregulated in TNBC. **A** Downregulated miRNAs in breast cancer tissue especially in TNBC were obtained from TCGA-BRCA database. **B** Heatmap of different downregulated miRNAs expression between TNBC and normal breast tissue from TCGA-BRCA database. **C** MirPath v.3 GO Reverse Search was used to find DNA damage-related miRNAs. Four candidate miRNAs were chosen from overlapping the above four gene sets and we chose miR-26a-5p as a preferred target. **D** The expression of miR-26a-5p in breast cancer (red bar) was downregulated compared with normal tissues (blue bar) from TCGA-BRCA database. Its expression in TNBC (red bar) was downregulated compared with NTNBC (blue bar). **E** The expression of miR-26a-5p in breast cancer tissues (red bar) and matched normal tissues (blue bar) was measured by RT-qPCR. Its expression in TNBC (red bar) was downregulated compared with NTNBC (blue bar) in clinical tissues. **F** The expression of miR-26a-5p in breast cancer cell lines and human normal breast epithelial cell line MCF10A was measured by RT-qPCR. ***P < 0.001, ****P < 0.0001

To search the special dysregulated microRNA in TNBC, we accessed the miRNA-Seq data of breast cancer from The Cancer Genome Atlas (TCGA). According to the corresponding clinical information downloaded from UCSC, the 1078 samples of TCGA-BRCA were divided into cancer tissues and normal tissues and the other two groups as non-triple-negative breast cancer (NTNBC) and TNBC. By using several R-related tools, we, respectively, got the dysregulated microRNAs between cancer tissues and normal tissues, NTNBC and TNBC. In this article, we decided to explore potential anti-oncogene; therefore, we overlapped the downregulated gene sets. The result indicated that 68 genes were downregulated in breast cancer tissue, especially in TNBC (Fig. 1A). With a view to the detectable of downregulated microRNAs in cancer tissue, we ranked 68 genes in expression level and selected the top 10 genes to further study (Fig. 1B). It has been reported that microRNAs participated in various biological processes; however, DNA damage-related research only occupied a small part. Therefore, we tend to find DNA damage-related microRNAs to provide potential therapeutic targets. To screen the target genes, we used mirPath v.3 GO Reverse Search [29], a miRNA pathway analysis web server, to search DNA damage-related categories. GO: 0000077, GO: 0006977, GO: 0042769 were analyzed to find miRNAs sets. The three sets overlapped with the top 10 genes and then got 4 candidate miRNAs as let-7b-5p, miR-26a-5p, let-7a-5p and miR-22-3p (Fig. 1C). It has been reported that the let-7 family and miR-22 could regulate DDR, so we chose miR-26a-5p as a preferred target [30, 31]. To verify the expression of miR-26a-5p in TNBC, we examined it in the 51 paired cancer tissues and matched normal tissues which were collected from the hospital. We found that miR-26a-5p was downregulated in breast cancer, especially in TNBC, and it was consistent with the results obtained from the TCGA-BRCA database (Fig. 1D, E). Next, we compared the differential expressions between breast normal cell lines and two subsets breast cancer cell lines. Consistent with the previous result, miR-26a-5p was downregulated in breast cancer cell lines, especially in TNBC cell lines (Fig. 1F).

Taken together, we demonstrated that miR-26a-5p is downregulated in breast cancer particularly in TNBC and it may play a role in DNA damage.

### miR-26a-5p upregulation promotes the sensitivity of Cisplatin and DNA damage in TNBC cell lines

**Fig. 2:**
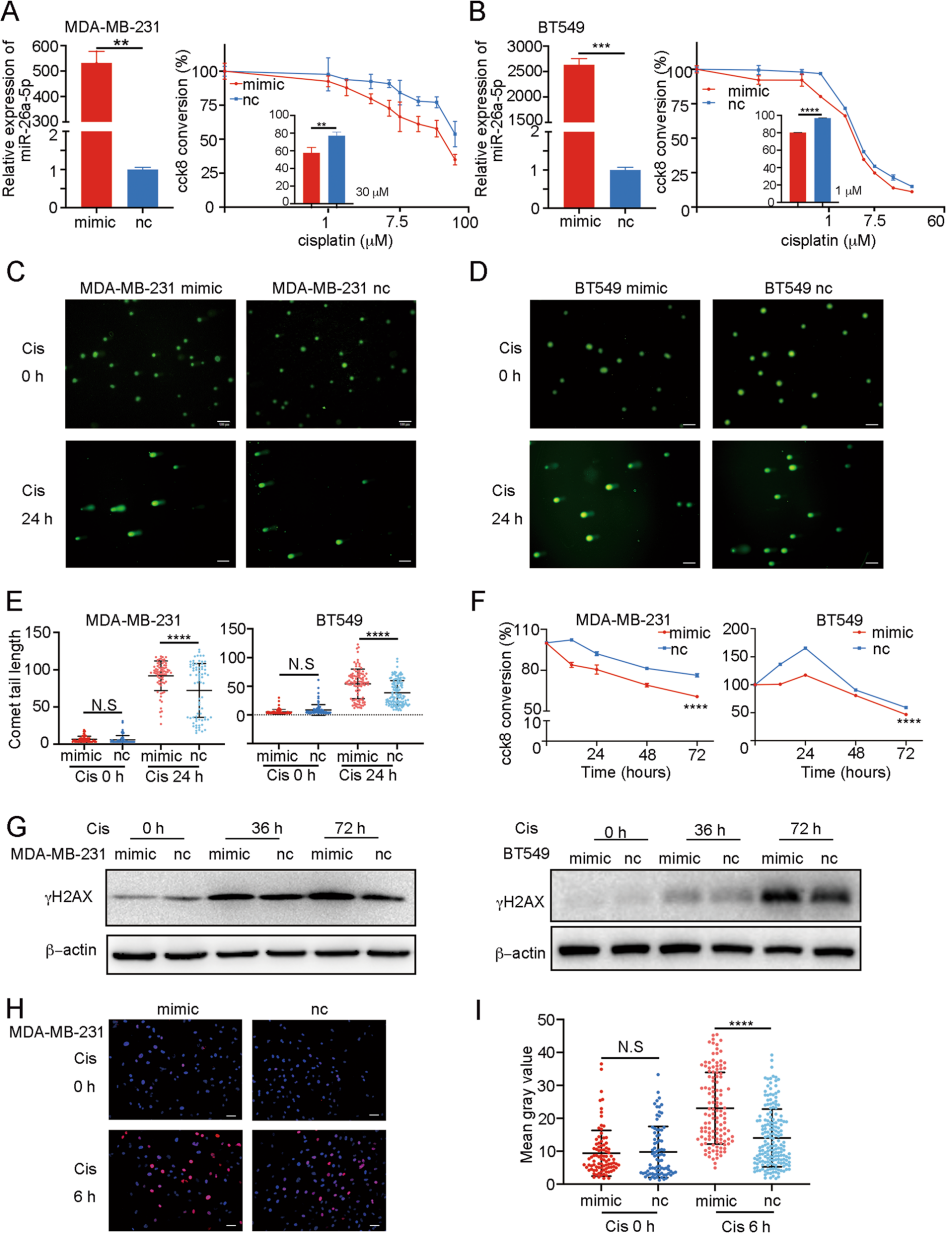
miR-26a-5p upregulation promotes the sensitivity of Cisplatin and DNA damage in TNBC cell lines. **A**, **B** Cisplatin-insensitive (MDA-MB-231) and Cisplatin-sensitive (BT549) TNBC cell lines were chosen to transfect miR-26a-5p mimics and normal control. Different concentrations of Cisplatin were diluted with medium containing 1% FBS to incubate cell lines for 72 h. CCK8 was used to detect cytotoxicity. **C–E** Comet assay was used to identify the level of DNA damage after Cisplatin treatment to MDA-MB-231 and BT549 cells overexpressing miR-26a-5p, respectively, in 20 µM and 2 µM. **F** CCK8 was used to detect the live cell count at different time points after a special concentration of Cisplatin treatment (MDA-MB-231, 20 µM and BT549, 2 µM). **G** Western blot was used to measure the expression of DNA damage mark, γH2AX, in MDA-MB-231 and BT549 cells overexpressing miR-26a-5p at different time points after Cisplatin treatment. **H**, **I** Immunofluorescence was adopted to detect γH2AX expression change in the MDA-MB-231 cells exposed to Cisplatin for 6 h. **P < 0.01, ***P < 0.001, ****P < 0.0001 vs. control. N.S. not significant. *n* = 3

Cisplatin interacts with DNA to induce DNA damage, therefore resulting in cell death. To assess the function of miR-26a-5p, we overexpressed miR-26a-5p by transfecting miR-26a-5p mimics in breast cancer cell lines. According to the Anne Margriet Heijink’s study, we chose Cisplatin-insensitive (MDA-MB-231) and Cisplatin-sensitive (BT549) TNBC cell lines, without BRCA1/2 mutation, to further study [32]. To avoid the proliferation of cancer cell lines influenced the detection of drug sensitivity, different concentrations of Cisplatin were diluted with medium containing 1% FBS to incubate cell lines for 72 h. We found that overexpression of miR-26a-5p promotes Cisplatin sensitivity in both MDA-MB-231 and BT549 cell lines (Fig. 2A, B). To verify whether miR-26a-5p plays a role in DNA damage, comet assay which is a sensitive tool to detect a single cell’s DNA damage was used to identify the level of DNA damage. And it showed that Cisplatin-induced comet tails in miR-26a-5p overexpression cells are significantly longer (Fig. 2C–E).

Next, we monitored the response to Cisplatin at different time points. We detected the live cell count by CCK8 at different time points after a special concentration of Cisplatin treatment. We found that MDA-MB-231 and BT549 cell lines exhibited different reaction capacity to Cisplatin. MDA-MB-231 showed quicker response to Cisplatin in the early stage and relatively weaker superposition in the later period. Interestingly, BT549 reacted to Cisplatin tardiness at the beginning till it woke up after 24 h. The discrepancy response to Cisplatin between the two cell lines may reflect different DNA damage response rates. Whereas, overexpressing miR-26a-5p in both cell lines stimulated the response to Cisplatin significantly at an early stage (Fig. 2F). Besides, we tested the γH2AX at different time points after treatment with Cisplatin. Corresponding to the timeline detection of Cisplatin, the MDA-MB-231 cell line appeared intensive DNA damage earlier than BT549. And beyond that, the accumulation effect of BT549 in the later treatment stage was more powerful. Likewise, both groups showed higher expression of γH2AX especially in miR-26a-5p-overexpressed groups after Cisplatin treatment (Fig. 2G). To precisely monitor DNA damage at an early stage of Cisplatin exposure, we adopted immunofluorescence to detect γH2AX expression change in the MDA-MB-231 cells which were exposed to Cisplatin for 6 h. We found that overexpressed miR-26a-5p accelerated γH2AX to DNA damage sites (Fig. 2H–I).

In summary, miR-26a-5p promoted DNA damage, thus enhancing the Cisplatin sensitivity.

### miR-26a-5p upregulation promotes apoptosis caused by Cisplatin-induced DNA damage

**Fig. 3:**
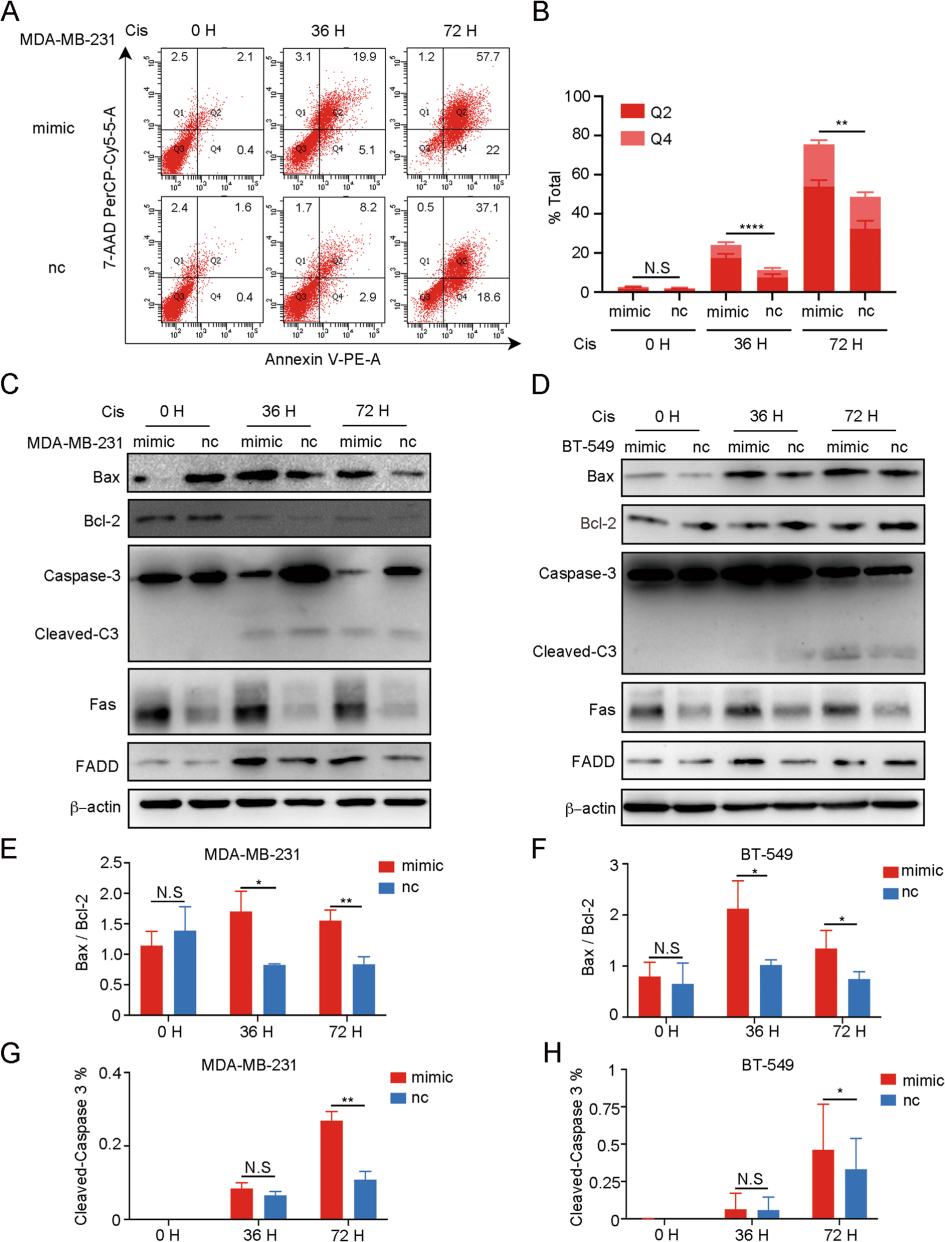
miR-26a-5p upregulation promotes apoptosis caused by Cisplatin-induced DNA damage. **A**, **B** Flow cytometry was used to detect early apoptosis and late apoptosis in the MDA-MB-231 cells overexpressing miR-26a-5p after Cisplatin exposure at different time points. **C**, **D** The expression of apoptosis markers in MDA-MB-231 and BT549 cells overexpressing miR-26a-5p after Cisplatin exposure was measured by Western blot. **E–H** The gray values were detected by Image J and then got the gray values ratio. **P < 0.01, ***P < 0.001, ****P < 0.0001 vs. control. N.S not significant. *n* = 3

Cisplatin-induced DNA damage fails to repair, and it will trigger apoptosis and end with cell death. Therefore, we detected the cells’ apoptosis level after Cisplatin exposure. Flow cytometry was used to detect early apoptosis and late apoptosis in the MDA-MB-231 cell line. The result indicated that miR-26a-5p enhanced Cisplatin-induced apoptosis in both stages (Fig. 3A, B) and beyond that we monitored apoptosis-related protein change in 0 H, 36 H and 72 H after Cisplatin treatment in MDA-MB-231 and BT549 cell lines. We found that miR-26a-5p enhanced the general apoptosis level (Fig. 3C–F). Besides, miR-26a-5p promoted cleaved Caspase-3 from Caspase-3 in both cell lines though MDA-MB-231 reacted earlier than BT549 (Fig. 3G, H). For the death receptor apoptosis pathway, we surprisingly found that miR-26a-5p improved Fas expression without Cisplatin and FADD with Cisplatin (Fig. 3C, D). It suggested that miR-26a-5p promoted Cisplatin-induced apoptosis through the mitochondrial pathway and the Fas death receptor pathway.

### miR-26a-5p directly targets the 3’UTR of BARD1 and NABP1

**Fig. 4:**
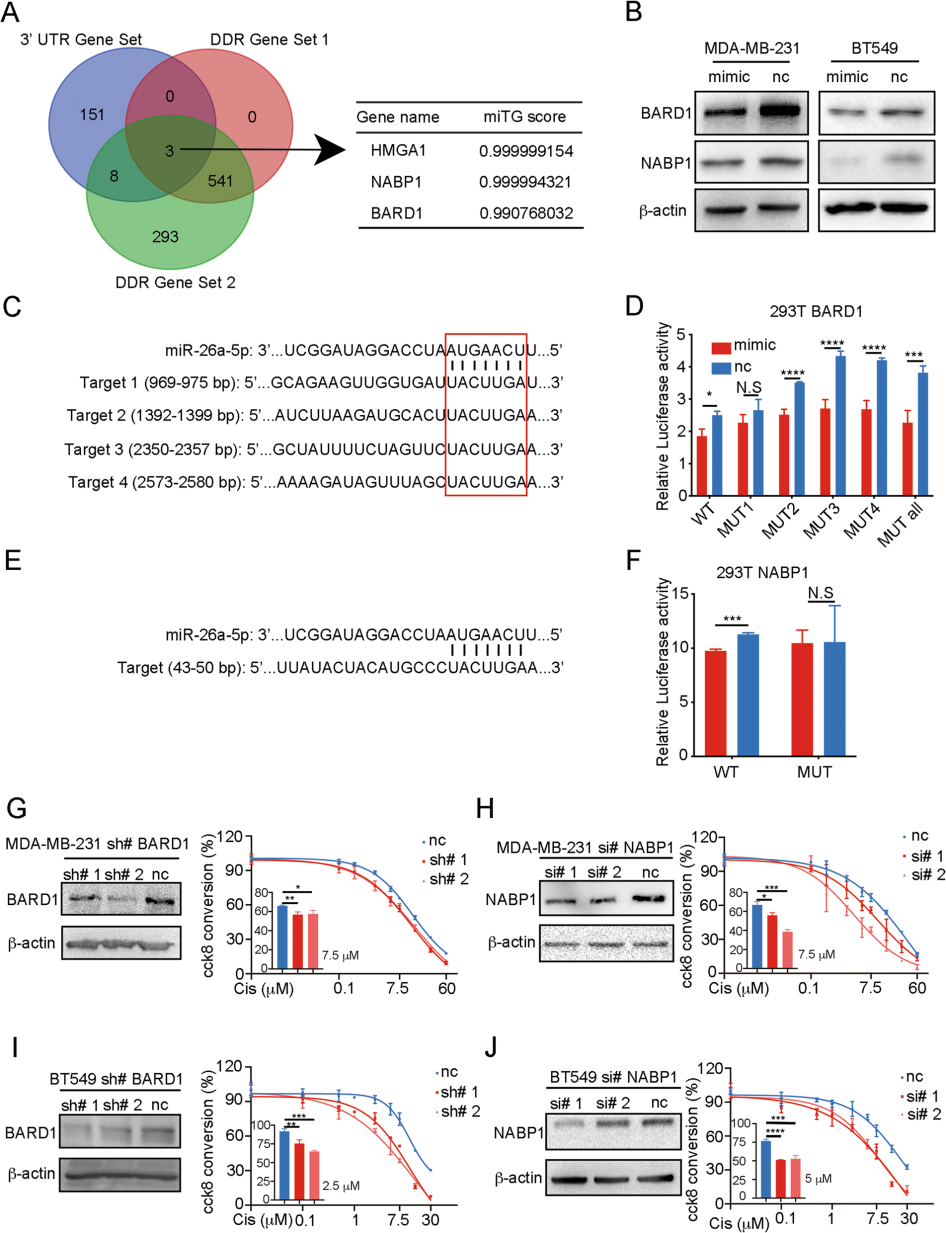
miR-26a-5p directly targets the 3’UTR of BARD1 and NABP1. **A** MicroT-CDS software was used to predict the downstream gene of miR-26a-5p. DDR gene sets were selected from GSEA randomly and then overlapped the three gene sets and got three potential target genes, HMGA1, NABP1 and BARD1. **B** BARD1 and NABP1 proteins were restrained after overexpressing miR-26a-5p in MDA-MB-231 and BT549 cells measured by Western blot. **C**, **D** TargetScan website was used to predict binding sites of miR-26a-5p and BARD1 mRNA. Dual-luciferase reporter assay was performed to confirm binding sites in 293T cells. **E**, **F** TargetScan website was used to predict binding sites of miR-26a-5p and NABP1 mRNA. Dual-luciferase reporter assay was performed to confirm binding sites in 293T cells. **G–J** CCK8 was used to measured cytotoxicity of Cisplatin after knockdown BARD1 in shRNA and NABP1 in siRNA in TNBC cells, respectively. *P < 0.05, **P < 0.01, ***P < 0.001, ****P < 0.0001 vs. control. N.S not significant. *n* = 3

The above results suggested that miR-26a-5p promoted DNA damage or impaired DNA damage repair (DDR). Given miRNAs were mostly confirmed to negatively regulate the expression of a target gene through binding 3’ UTR of mRNA to repress translation. We hypothesized that miR-26a-5p may downregulate the expression of the DDR genes to promote DNA damage. To search the target gene of miR-26a-5p, we took three steps as follow: firstly, predicting the downstream genes by MicroT-CDS which was a software from DINAN TOOLS with miTG score > 0.99 and got the first gene set [33]. DDR gene sets were selected from GSEA randomly. Finally, we overlapped the gene sets and got three potential target genes which were HMGA1, NABP1 and BARD1 (Fig. 4A). HMGA1 had been verified that miR-26a-5p binds to its’ 3'UTR mRNA [34], and therefore, we chose BARD1 and NABP1 as candidate downstream genes. Overexpressed miR-26a-5p in TNBC cell lines and we found that both BARD1 and NABP1 protein were restrained (Fig. 4B). Predicted binding sites were accessed from the TargetScan website, and a dual-luciferase reporter assay was performed to confirm binding sites. The 3'UTR of BARD1 mRNA was predicted 4 potential binding sites, respectively, located in 969–975 bp, 1392–1399 bp, 2350–2357 bp, 2573–2580 bp. And only when the first predicted binding site was mutated, there was no significant difference in relative luciferase activity between the groups. It suggested that miR-26a-5p bound with 3'UTR of BARD1 mRNA at 969–975 bp (Fig. 4C, D). Similarly, the luciferase reporter activity was recovered when the predicted binding site of NABP1 was mutant, locating at 43–50 bp of 3’ UTR (Fig. 4E, F).

In general, miR-26a-5p regulated the expression of BARD1 and NABP1 protein by binding to the 3’ UTR at specific sites and restrained their translation.

### miR-26a-5p promotes Cisplatin-induced cell death via BARD1 and NABP1

**Fig. 5:**
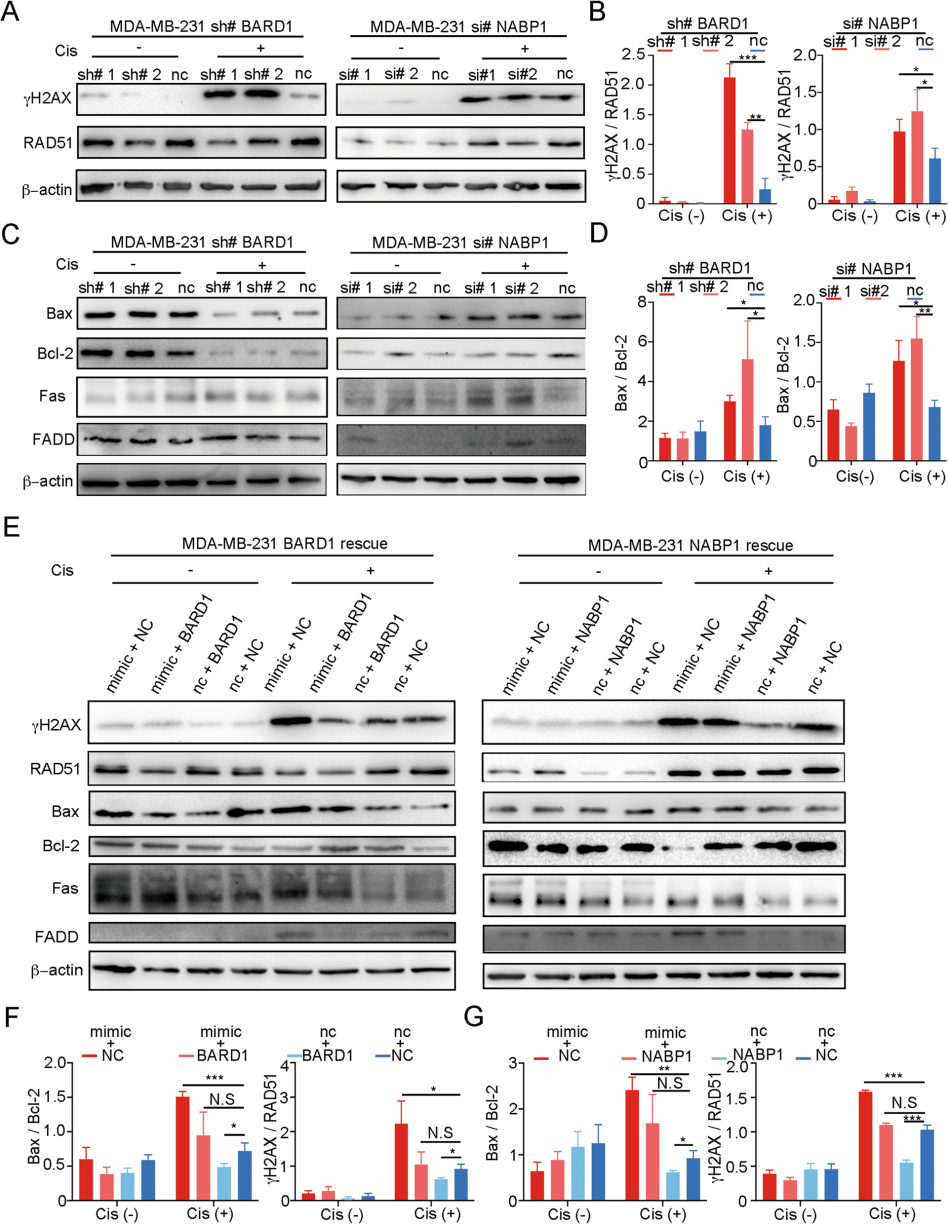
miR-26a-5p promotes Cisplatin-induced cell death via BARD1 and NABP1. **A**–**D** MDA-MB-231 cells were knockdown BARD1 in shRNA and NABP1 in siRNA, respectively. DNA damage marker (γH2AX), DDR marker (RAD51) and apoptosis markers were detected without or with 20 µM Cisplatin stimulating by Western blot. **E–G** BARD1 or NABP1 was transfected into miR-26a-5p-overexpressing MDA-MB-231 cells. Western blot was performed to evaluate the expression of the markers with or without Cisplatin stimulating. The gray values were detected by Image J and then got the gray values ratio. *P < 0.05, **P < 0.01, ***P < 0.001, ****P < 0.0001 vs. control. N.S not significant. *n* = 3

To examine whether BARD1 and NABP1 enhance Cisplatin sensitivity, MDA-MB-231 and BT549 cell lines were, respectively, transduced with small interference RNAs (siRNAs) of NABP1 and short hairpin RNAs (shRNAs) of BARD1. Consistent with our prediction, knockdown of them results in enhanced Cisplatin sensitivity in both cell lines (Fig. 4G–J). To test whether the promoted sensitivity of Cisplatin was caused by HR defects (HRD), we analyzed RAD51 protein expression after Cisplatin treatment as an index of HR proficiency in MDA-MB-231 cell line. When γH2AX and RAD51 were compared simultaneously, it was better represented the dynamics of DNA damage and DDR. We found that knockdown of BARD1 or NABP1 displays a relatively stronger DNA damage and weaker DDR in MDA-MB-231 (Fig. 5A, B). To analyze the apoptosis signaling change after Cisplatin exposure, we test Bax, Bcl-2, Fas, FADD protein expression. After treatment with Cisplatin for 72 h, knockdown of BARD1 or NABP1 in MDA-MB-231 cells showed more active apoptosis with a relatively higher expression level of Bax and lower Bcl-2, comparing with normal control. Interestingly, when BARD1 or NABP1 was inhibited, the expression level of Fas and FADD were not significantly changed without Cisplatin treatment. However, both of Fas and FADD were enhanced when the cell lines were exposed to Cisplatin (Fig. 5C, D). It suggested that BARD1 or NABP1 did not affect Fas or FADD’s expression in a normal situation. Increasing DNA damage upregulates Fas and FADD with Cisplatin treatment after knockdown of BARD1 or NABP1.

To further confirm that miR-26a-5p functioned as a cell death warrant after Cisplatin treatment via BARD1 and NABP1, we performed a rescue assay, respectively, in the MDA-MB-231 cell line. We found that simultaneously overexpress miR-26a-5p and BARD1 recede the DNA damage dynamic compared with overexpressing miR-26a-5p alone. And similar results were shown in the NABP1 overexpression cells. Moreover, overexpressing BARD1 or NABP1 suppressed the promotion of apoptosis by miR-26a-5p with Cisplatin treatment in general. However, the expression of Fas was not changed by overexpressing BARD1 or NABP1 in miR-26a-5p-overexpressed cell lines without Cisplatin. It suggested that miR-26a-5p enhanced Fas via other mechanisms rather than BARD1 or NABP1 in normal conditions. When cells were treated with Cisplatin, overexpressing BARD1 or NABP1 rescued miR-26a-5p-induced Fas promotion and we attributed it to intensive DNA damage (Fig. 5E–G).

Collectively, the results suggested that miR-26a-5p impaired DDR and relatively strengthened DNA damage via negatively regulating BARD1 and NABP1. Therefore, it enhanced the general apoptosis by strengthening Cisplatin-induced DNA damage. It was worth noting that miR-26a-5p positively regulated Fas which may create a hypersensitive state of death receptor pathway regulated apoptosis.

### In vivo effect of miRNA-26a-5p on the Cisplatin sensitivity

**Fig. 6:**
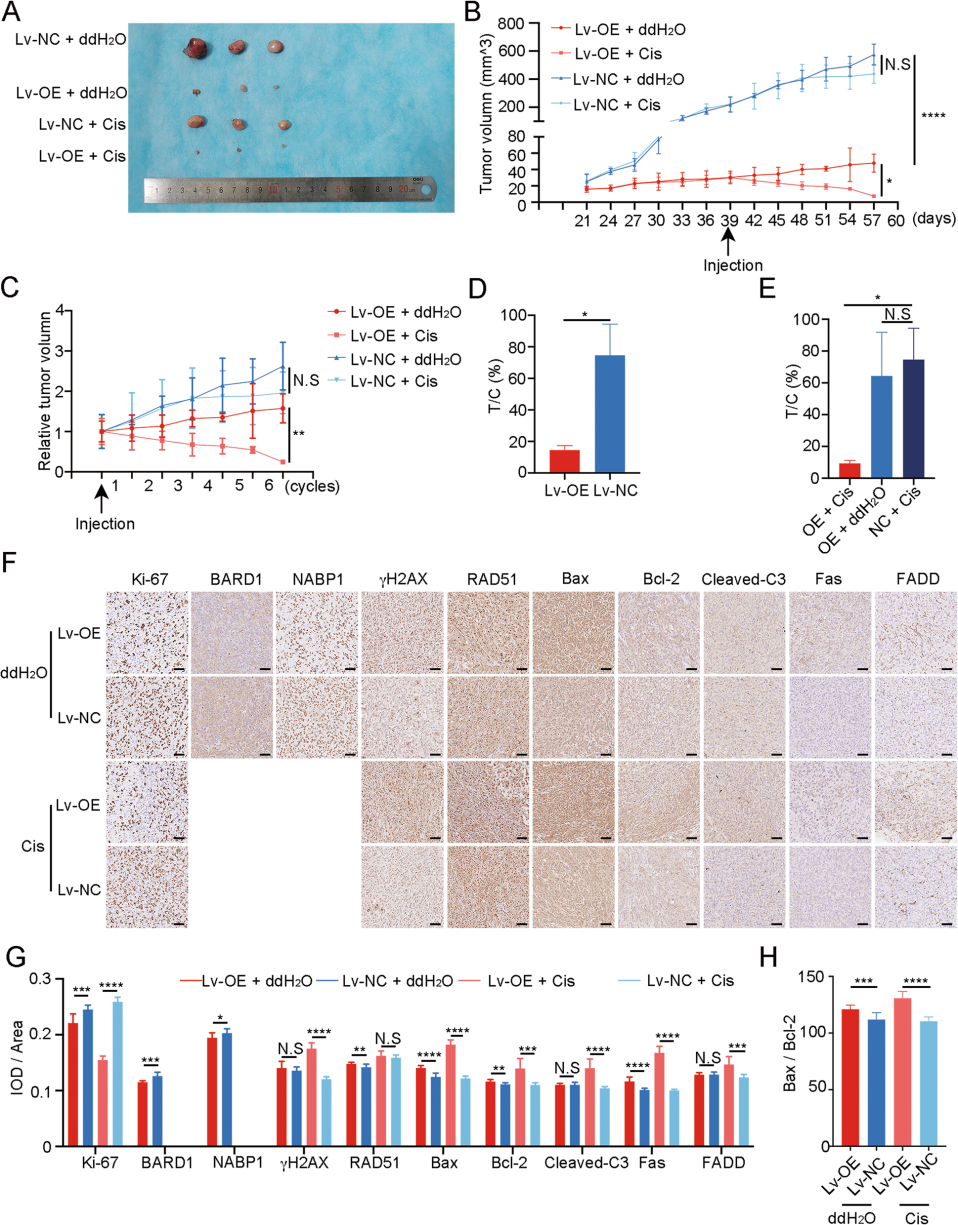
In vivo effect of miRNA-26a-5p on the Cisplatin sensitivity. **A**, **B** The indicated cells were implanted orthotopically into the second mammary fat pad (i.m.f.p.) of female BALB/C nude mice. The volumes of subcutaneous tumors in the indicated groups (*n* = 3) were recorded. **C** RTV was introduced to illustrate Cisplatin effect on Cisplatin sensitivity in vivo*.* **D**, **E** T/C% was introduced to evaluate drug efficacy in vivo. **F–H** Representative images of IHC staining and the relative scores (*n* = 12) of the indicated markers in subcutaneous tumors of mice. *P < 0.05, **P < 0.01, ***P < 0.001, ****P < 0.0001 versus control. N.S not significant

To verify the role of miR-26a-5p in vivo in Cisplatin treatment, MDA-MB-231/Lv-overexpressed (Lv-OE) and MDA-MB-231/Lv-control (Lv-NC) cells mixed with matrigel were implanted orthotopically into the second mammary fat pad (i.m.f.p.) of female BALB/C nude mice. After 21 days’ growth, the volumes of subcutaneous tumors were recorded. Peritoneal injection with Cisplatin (2.5 mg/kg) or ddH_2_O was used when one group’s volume exceeded 150 mm^3^. And the treatment lasted for 6 cycles, 3 days interval between each cycle. We found that miR-26a-5p had a significant negative effect on tumor growth (Fig. 6A). Besides, after Cisplatin treatment, the Lv-OE group reacted to the drug at the end of the first cycle and the tumor volume continued to decrease. The Lv-NC group did not response until the fourth cycle following a flat state (Fig. 6B). To better illustrate its effect on Cisplatin sensitivity in vivo, we introduced the relative tumor volume (RTV) and T/C%. The RTV could be more visualized to exhibit the response of each group to medication. As shown in the panel, the RTV of the Cisplatin-treated Lv-OE group decreased as the treatment cycles progressed (Fig. 6C). At the end of the treatment cycle, we calculated the terminal volume as T/C%. However, evaluation criteria for drug efficacy as follow: T/C% > 60% is invalid; T/C% < 60% and *P* < 0.05 is valid [35]. Obviously, T/C_Lv-OE_% was less than 60% with *P* < 0.05 and T/C_Lv-NC_% was more than 60% (Fig. 6D). If we also regard overexpressing miR-26a-5p as a therapy, it had no significant difference between T_Lv-OE+ddH2O_/C_Lv-NC+ddH2O_% and T_Lv-NC+CIS_/C_Lv-NC+ddH2O_%. Nevertheless, when miR-26a-5p was combined with Cisplatin, it exhibited a striking effect on anti-tumor (Fig. 6E). Hence, miR-26a-5p combined with Cisplatin could represent synthetic lethality.

Furthermore, IHC was used to detect protein expression in solid tumors which were removed from the subcutaneous tumor model. Ki-67 was detected to exhibit the negative effect of miR-26a-5p on tumor growth, especially under Cisplatin exposure. Besides, miR-26a-5p did repress the expression of BARD1 and NABP1 which were in line with the previous results. When the Lv-OE group was treated with Cisplatin, the expression of γH2AX was significantly higher than the Lv-NC group. However, RAD51 had no significant difference. It suggested that miR-26a-5p enhanced Cisplatin-induced DNA damage in vivo. In terms of apoptosis, the Lv-OE group represented more active Bax, Bcl-2 and Cleaved-C3 than the Lv-NC group with Cisplatin treatment. To show the total apoptotic state more directly, we used the Bax/Bcl-2 ratio. And it implicated that miR-26a-5p may boost apoptotic state in vivo. Apart from that, the Lv-OE group showed higher expression of Fas with or without Cisplatin stimulation compared to the Lv-NC group. However, the expression of FADD improved in the Lv-OE group when treat with Cisplatin (Fig. 6F–H).

To sum up, miR-26a-5p inhibited tumor growth and enhanced the sensitivity of Cisplatin in vivo.

### miR-26a-5p upregulation promotes the sensitivity of Olaparib and drug combination

**Fig. 7:**
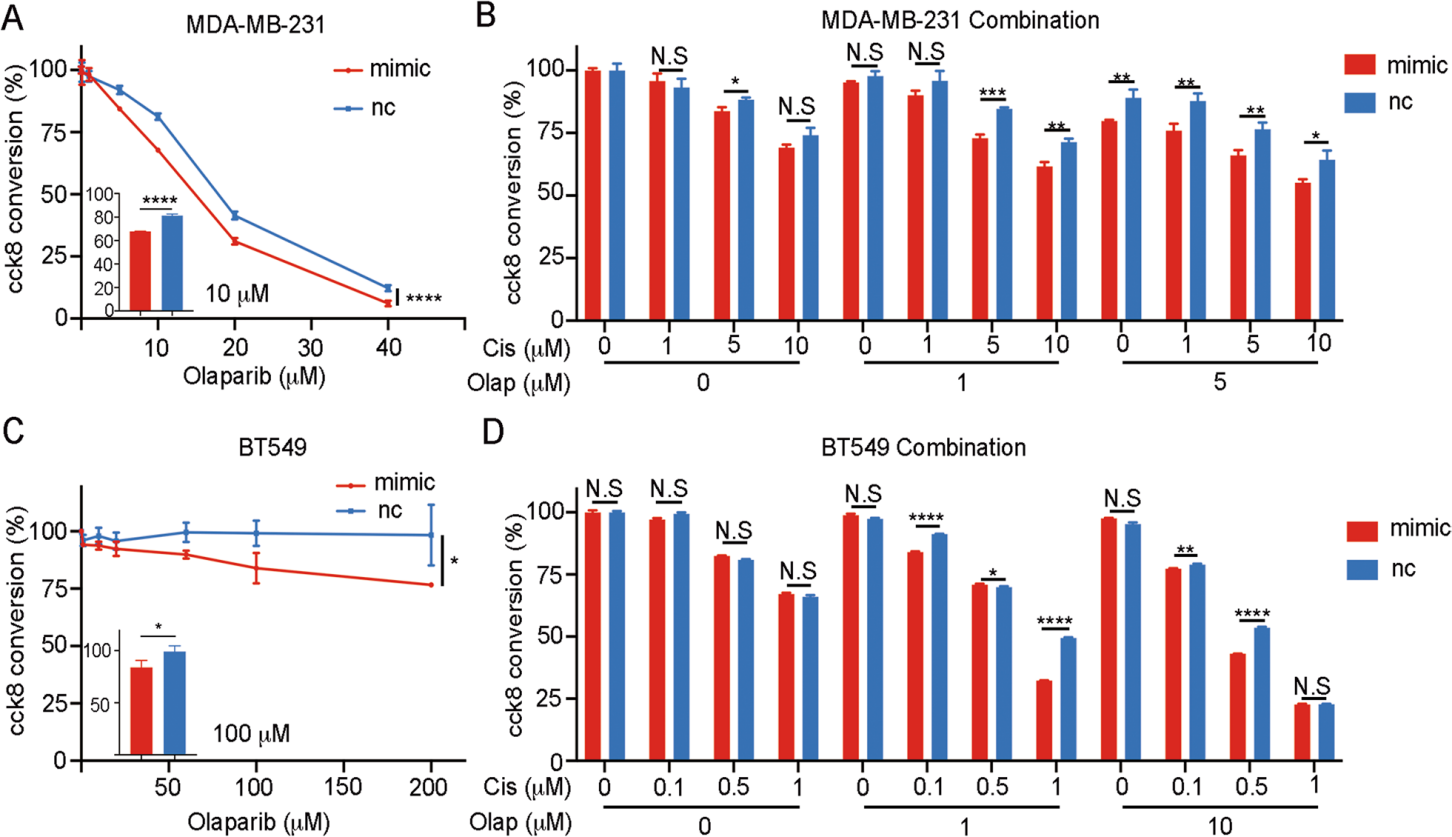
miR-26a-5p upregulation promotes the sensitivity of Olaparib and drug combination. **A** MDA-MB-231 cells were transfected with miR-26a-5p mimics and normal control. Different concentrations of Olaparib were diluted with medium containing 1% FBS to incubate cell lines for 72 h. CCK8 was used to detect cytotoxicity. **B** CCK8 was used to detect cytotoxicity of the drug combination of Cisplatin and Olaparib in MDA-MB-231 overexpressing miR-26a-5p and normal control. **C**, **D** CCK8 was used to detect the indicated drug effect on BT549 cells. *P < 0.05, **P < 0.01, ***P < 0.001, ****P < 0.0001 vs. control. N.S not significant. *n* = 3

The previous results indicated that miR-26a-5p negatively regulate HR thus contributing to HRD and it may act as BRCAness. As the new definition of BRCAness suggested that it was a DDR phenocopy loss of BRCA1 or BRCA2 to form HRD, which may be sensibilization to DNA damaging agents and PARPis, ultimately leading to synthetic lethality with PARPis [25]. To further confirm that it acts as BRCAness, we introduced Olaparib, a PARPi, to treat breast cancer cells and evaluated its cytotoxicity. As shown in the figure, we found that miR-26a-5p promoted Olaparib sensitivity in both cell lines. And BT549 cell line showed higher resistance to Olaparib (Fig. 7A, C). Next, we wondered whether miR-26a-5p affects the drug combination of Cisplatin and Olaparib. Different concentrations of two drugs were mixed together to incubate with cells for 72 h. At the special concentration ratios, miR-26a-5p significantly enhanced the sensitivity of drug combinations (Fig. 7B, D). In short, miR-26a-5p could act as BRCAness.

### miR-26a-5p is a transcriptional downstream target of ER-a and PR

**Fig. 8:**
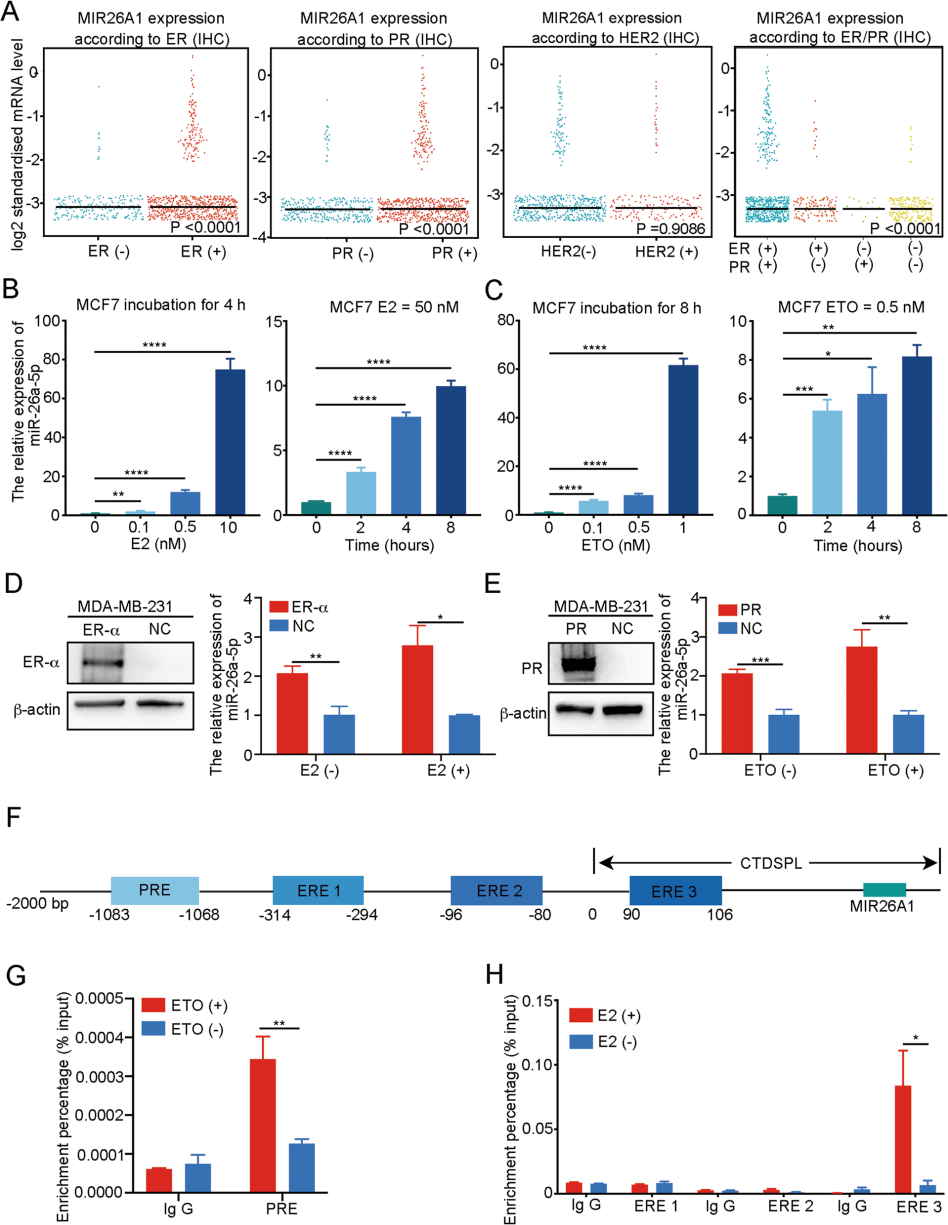
miR-26a-5p is a transcriptional downstream target of ER-a and PR. **A** bc-GenExMiner was used to analyze the expression level of miR-26a-5p which was positively correlated with ER-a status and PR status, not HER2 receptor status. **B** After hormone deprivation for 48 h, MCF-7 cells were stimulated with β-Estradiol (E2). RT-qPCR was used to monitor the expression of miR-26a-5p in different concentration or at different time points. **C** After hormone deprivation for 48 h, MCF-7 cells were stimulated with Etonogestrel (ETO). RT-qPCR was used to monitor the expression of miR-26a-5p in different concentration or at different time points. **D, E** Overexpressed ER-a or PR plasmid were transfected into MDA-MB-231 cells. RT-qPCR was used to detect the expression of miR-26a-5p in normal culture conditions without or with extra hormone stimulating. **F** JASPAR and hTFtarget databases were used to predict the potential binding sites of ER-a and PR with the promoter of miR-26a-5p. **G, H** ChIP assay was used to verify the binding sites. *P < 0.05, **P < 0.01, ***P < 0.001, ****P < 0.0001 versus control. *n* = 3

To explore the reason that miR-26a-5p is downregulated in TNBC, we browsed the online tool called bc-GenExMiner [36–38]. Considering the data consistency, we selected the RNA-seq data from TCGA to maintain consistency with the first filtered database in our research. Then we performed an exhaustive expression analysis of miR-26a-5p. We found that its expression was positively correlated with ER-a status and PR status, not HER2 receptor status. Besides, ER-a and PR status combinations analysis suggested ER-a might play a stronger role compared with PR (Fig. 8A). Therefore, ER-a and PR may participate in the regulation of the expression of miR-26a-5p. In consideration of ER-a and PR where hormone-dependent receptor proteins also function as transcriptional factors, we used β-Estradiol (E2) and Etonogestrel (ETO) stimulation assay to detect the expression of miR-26a-5p in ER-a and PR positive breast cell lines, MCF-7 and T47D. After hormone deprivation by cell lines culturing with phenol red-free medium supplemented with 10% dextran charcoal-stripped bovine serum for 48 h, we stimulated cell lines with different concentrations of E2 and ETO, respectively, for different exposure times. After incubating the MCF-7 cell line with phenol red-free medium containing different concentrations of E2 for 4 h, qRT-PCR was used to monitor the expression of miR-26a-5p. It showed that the expression level raised with the increasing stimulus concentration. In addition, the medium included 50 nM E2 stimulated MCF-7 cell line for disparate time scale scales and we found that the expression of miR-26a-5p increased over time (Fig. 8B). And beyond that, the ETO stimulation assay had similar results that it promoted the expression of miR-26a-5p with the increase in effective concentration and extension of effective time span (Fig. 8C). Similarly, we obtained the same results in the T47D cell line (Additional file 1: Fig. S1). To further verify that ER-a and PR take part in the regulation of miR-26a-5p expression, we chose ER-a and PR negative breast cancer cell line MDA-MB-231 to overexpress ER-a and PR separately. We found that overexpressed ER-a or PR enhances the expression of miR-26a-5p in normal culture conditions without extra E2 or ETO. Apart from that, after hormone deprivation for 48 h, we cultured cells with phenol red-free medium included additional E2 or ETO. Equally unsurprisingly, E2 or ETO further increased miR-26a-5p expression (Fig. 8D, E).

Considering that they may regulate the expression of miR-26a-5p as transcription factors, we performed a ChIP assay to verify the assumption as following steps. Firstly, we got the promoter sequence of miR-26a-5p from the NCBI Gene. Accidentally, we found that the host gene of miR-26a-5p, MIR26A1, locates in the intron of the CTDSPL gene; therefore, we chose the 2000 bp of CTDSPL gene upstream to the 200 bp of downstream as a promoter sequence. Next, we took advantage of online tools to predict the potential binding sites on promoter sequence, such as JASPAR, a database of transcriptional factor binding profiles [39], and hTFtarget, a comprehensive database for regulations of human transcription factors and their targets [40]. The binding sites that ER-a combined with the promoter were predicted by the JASPAR database with relative score > 0.7; therefore, we got three sites as − 314 to − 294 bp, − 96 to − 80 bp, 90–106 bp. Furthermore, the binding sites of PR on promoter were predicted by hTFtarget as − 1083 to − 1068 bp. Finally, we correspondingly designed the primer sequence and conducted the ChIP assay in the MCF-7 cell line. The results suggested that ER-a combined with the promoter at 90–106 bp and PR’s site was at − 1083 to − 1068 bp (Fig. 8F–H).

## Discussion

Tumor heterogeneity and lack of effective treatment result in the worst outcomes of TNBC. Researches on tumor microenvironment and tumor immunity bring new strategy insights to TNBC therapy. However, chemotherapy remains the most important systemic treatment and the optimal integration of platinum drugs is still a controversial issue [41]. HRR plays a vital role in DDR, and the pathway-related genes could be the targets to invent innovative drugs. BARD1 cooperates with BRCA1 to form a heterodimer complex, triggering DSBs into the HR pathway for damage repair [42]. NABP1, a homolog of hSSB1, has been reported to play a role in the HR-dependent repair of DSBs [17]. Our results confirmed that miR-26a-5p impaired HRR by negatively regulating BARD1 and NABP1. A new definition of BRCAness revealed that it formed HRD, thus sensibilizing to DNA damaging agents and PARPis, ultimately leading to synthetic lethality with PARPis [25]. PARP1is inhibit DNA repair through PARP1 trapping, thus promoting sensitivity of radiotherapy and chemotherapy drugs. Besides, in BRCA-mutated cancers, the deficient of PARP1 could lead to synthetic lethality. Phase 3 OlympiA clinical trial for Olaparib has demonstrated that patients with germline BRCA1 or BRCA2 pathogenic or likely pathogenic variants could obtain a benefit in early breast cancer [43]. To further illustrate miR-26a-5p acts as BRCAness, we verified its sensibilization about Olaparib, a PARP1 inhibitor. Therefore, the miR-26a-5p/BARD1/NABP1 axis may alter HRR function to some degree and result in BRCAness with enhanced genomic instability, leading to the sensitivity to Cisplatin, Olaparib and their combination. Interestingly, in our study, we found that MDA-MB-231 and BT549 showed significantly different sensitivity to Olaparib and reversed to Cisplatin. Both cell lines are BRCA1/2 wild TNBC cell lines. However, MDA-MB-231 exhibits more sensitivity to Olaparib than BT549. It may suggest that there are stronger regulators expect BRCA1 or BRCA2 to modulate response to Olaparib. Next step, we will explore more potential molecular for PARPis therapy.

Recent studies have revealed the emerging role of miRNAs in cancer treatment, such as involvement in the tumor immune escape and interaction with TME. In addition, miRNAs’ dysregulation participates in the regulation of DDR and drugs’ response. For instance, the miR-302 family is reported that they enhance breast cancer cells’ sensitivity to radiotherapy [44]. It is worth noting that the third medical revolution led by nucleic acid drugs is getting increasingly hot. Epigenetic modifications include miRNAs regulation rising as a new direction of cancer therapy. Therefore, miRNA-based therapeutics via targeting oncomiRNAs or restoring anti-oncomiRNAs are innovative. According to P. Mondal et al. review, there are numerous anti-miRNAs and miRNA mimics related to cancer under preclinical studies or clinical trials [45]. For example, MesomiR-1, a mimic of miR-16, is packed in EDV™ nanocells to target EGFR. It has been demonstrated that MesomiR-1 exhibited well in Phase I [46]. Apart from that, Wenqiang Yu et al. hold the view that reactive dysfunction of tumor suppressor genes by enhancer switching through NamiRNA network may be a potential treatment strategy [47]. In our research, we found that miR-26a-5p promoted Cisplatin sensitivity for the first time. In vivo, we demonstrated that miR-26a-5p overexpression inhibited tumor growth and improved the curative effect of Cisplatin. Hence, miR-26a-5p is a potential target to achieve miRNA-based therapeutics. Although limitations in miRNA-based therapeutics are their physicochemical characteristics which influence their efficiencies, chemical modification of miRNAs and innovative delivery methods may expand the application of the therapy.

Cisplatin-induced cell death remains several steps: Firstly, it causes DDR. Then, mitochondrial outer membrane permeabilization would trigger intrinsic apoptosis and other components of the extrinsic apoptotic pathway [11]. Fas receptor-dependent pathway and mitochondrial-dependent pathway have been verified to take part in Cisplatin-induced cell death. It has been reported that Cisplatin promotes Fas death receptor pathway apoptosis independent of Fas ligand in human colon cancer cells. It suggested that chemotherapeutic agents could activate the Fas receptor and recruit FADD [48]. In our research, we found that miR-26a-5p promotes Cisplatin-induced apoptosis both in the mitochondrial-dependent pathway and Fas death receptor pathway. Interestingly, miR-26a-5p could enhance the expression of Fas under normal situations. It indicated that miR-26a-5p may build a hypersensitivity of the Fas death receptor state. However, MMR defects and other DDR defects induce the expression of FAS, FASL and TRAILR death receptors at the tumor cell surface [49]. It suggested that miR-26a-5p could regulate Fas expression via other DDR defects. It possibly brings a deeper insight into the function of miR-26a-5p.

Dysregulation of gene expression usually happens at the following levels: gene mutation, transcriptional regulation, post-transcriptional regulation, translational regulation and post-translational regulation. In this study, we found that miR-26a-5p is downregulated in breast cancer, especially in TNBC. To figure out the upstream factors downregulating miR-26a-5p in TNBC, we assumed it correlated with ER-a and PR status. ChIP assay confirmed that ER-a and PR regulate its expression in at the transcriptional level. However, it needs further study to investigate whether other mechanism, as DNA promoter methylation or gene mutation, involves in regulation of miR-26a-5p.

## Conclusions

In summary, we found that miR-26a-5p is downregulated in breast cancer, especially in TNBC. ER-a and PR promoted transcription and resulted in the different expression levels between TNBC and NTNBC. Besides, miR-26a-5p enhanced HRD via negatively regulating BARD1 and NABP1 which lead to improve the sensitivity to DNA damage agents and synthetic lethal. Furthermore, miR-26a-5p also reinforced apoptosis in the Fas death receptor pathway with or without Cisplatin and mitochondrial-dependent pathway with Cisplatin. Therefore, miR-26a-5p may act as a new BRCAness to offer chemotherapy decisions and provide a new target for miRNA-based therapeutics.

## Supplementary Information

**Additional file 1: Figure S1** Hormone stimulation assay in T47D cells.**A** After hormone deprivation for 48 h, T47D cells were stimulated with β-Estradiol. RT-qPCR was used to monitor the expression of miR-26a-5p in different concentration or at different time points. **B** After hormone deprivation for 48 h, T47D cells were stimulated with Etonogestrel. RT-qPCR was used to monitor the expression of miR-26a-5p in different concentration or at different time points. **P < 0.01, ***P < 0.001, ****P < 0.0001 versus control. N.S. not significant. *n* = 3.

## Data Availability

All data in our study are available upon request.

## Abbreviations

DDR: DNA damage repair (DDR)
TNBC: Triple-negative breast cancer
RT-qPCR: Quantitative reverse transcription polymerase chain reaction
ChIP: Chromatin immunoprecipitation
HRD: Homologous recombination repair defect
UTR: Untranslated region
ICLs: Intra- and inter-strand DNA cross-links
ssDNA: Single-strand DNA
DSBs: Double-strand breaks
NER: Nucleotide excision repair
MMR: Mismatch repair
HR: Homologous recombination
NHEJ: Non-homologous end joining
HRR: HR repair
gBRCAm: Germline BRCA1/2 mutations
PARPis: Poly-(ADP ribose)-polymerase inhibitors
TCGA: The Cancer Genome Atlas
siRNAs: Small interference RNAs
shRNAs: Short hairpin RNAs
RTV: Relative tumor volume
E2: β-Estradiol
ETO: Etonogestrel
