# Supplementary material for: Transcriptionally regulated miR-26a-5p may act as BRCAness in Triple-Negative Breast Cancer
Source: Breast Cancer Res. 2023 Jun 26;25:75. doi: 10.1186/s13058-023-01663-y (PMC10294332; doi:10.1186/s13058-023-01663-y)
Supplement: Supplementary file 1 — Additional file 1: Figure S1 Hormone stimulation assay in T47D cells.A After hormone deprivation for 48 h, T47D cells were stimulated with β-Estradiol. RT-qPCR was used to monitor the expression of miR-26a-5p in different concentration or at different time points. B After hormone deprivation for 48 h, T47D cells were stimulated with Etonogestrel. RT-qPCR was used to monitor the expression of miR-26a-5p in different concentration or at different time points. **P < 0.01, ***P < 0.001, ****P < 0.0001 versus control. N.S. not significant. n = 3. [file 13058_2023_1663_MOESM1_ESM.doc]

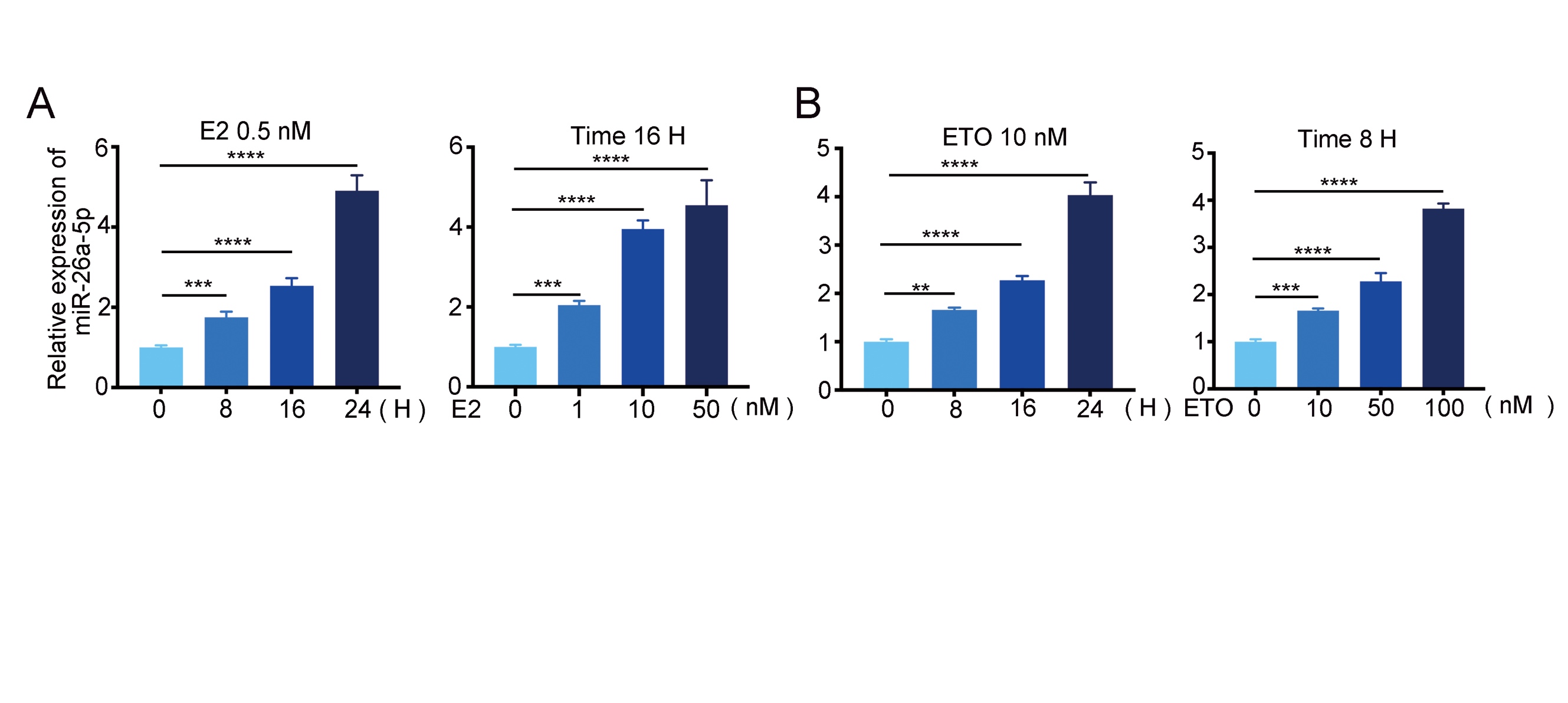


**Supplementary Figure 1.** **Hormone stimulation assay in T47D cells.**

**A** After hormone deprivation for 48 h, T47D cells were stimulated with β-Estradiol (E2). RT-qPCR was used to monitor the expression of miR-26a-5p in different concentration or at different time points. **B** After hormone deprivation for 48 h, T47D cells were stimulated with Etonogestrel (ETO). RT-qPCR was used to monitor the expression of miR-26a-5p in different concentration or at different time points. ***P* < 0.01, ****P* < 0.001, *****P* < 0.0001 vs. control. N.S. not significant. n = 3.
